# Evaluation of the Effect of Chairside Adjustment Extent on the Biaxial Flexural Strength of Aged Monolithic Zirconia

**DOI:** 10.7759/cureus.94829

**Published:** 2025-10-17

**Authors:** Nafise Elmamooz, Zahra Doosty

**Affiliations:** 1 Department of Restorative Dentistry, School of Dentistry, Kerman University of Medical Sciences, Kerman, IRN

**Keywords:** aging, biaxial flexural strength, chairside adjustment, monolithic, zirconia

## Abstract

Background and objective

Chairside adjustment of monolithic zirconia restorations is a common clinical procedure. This study aimed to investigate how surface treatment procedures and the extent of adjustment affect the biaxial flexural strength (BFS) of monolithic zirconia after artificial aging.

Methods

Fifty-six disk-shaped specimens (Ø15 × 1.2 ± 0.2 mm) were milled, sintered, and glazed from zirconia blocks (Ceramill, Amann Girrbach, Koblach, Austria) according to ISO 6872:2024. The specimens were then divided into seven groups (n = 8) based on surface treatment (grinding, polishing, or reglazing) and extent of adjustment (one vs. two areas): no adjustment (G1), grinding one area (G2), grinding + polishing one area (G3), grinding + reglazing one area (G4), grinding two areas (G5), grinding + polishing two areas (G6), and grinding + reglazing two areas (G7). All specimens were hydrothermally aged in an autoclave (134 °C, 2 bar, five hours) before BFS testing (piston-on-three-ball method, crosshead speed 1 mm/min). Data were analyzed using two-way ANOVA and Tukey’s post hoc test (α = 0.05).

Results

The highest BFS value was recorded for group G6 (grinding + polishing two areas), whereas group G4 (grinding + reglazing one area) showed the lowest value. Both surface treatment and extent of adjustment significantly affected the BFS of monolithic zirconia specimens (p < 0.05). Grinding and polishing significantly increased BFS values, while reglazing had a significant negative effect.

Conclusions

Proper polishing after chairside grinding provided the best flexural strength following artificial aging, whereas reglazing may compromise the flexural strength of monolithic zirconia. The flexural strength of monolithic zirconia is significantly influenced by the extent of adjustment.

## Introduction

Yttria-stabilized tetragonal zirconia polycrystal (Y-TZP) dental ceramic, known for its excellent mechanical and esthetic properties, is widely used in various clinical applications [1-3]. Zirconia is a polymorphic material that exists in three phases depending on temperature: monoclinic (m) from room temperature up to 1700 °C, tetragonal (t) between 1700 and 2370 °C, and cubic above 2370 °C until melting. To maintain the tetragonal phase at room temperature, stabilizing oxides such as yttrium oxide are added. Y-TZP exhibits high strength and fracture toughness due to its resistance to crack propagation [4].

Y-TZP has been used as a substructure for fixed partial dentures (FPDs) veneered with porcelain. Despite the superior esthetics of bilayered porcelain-fused-to-zirconia (PFZ) structures, the high incidence of veneer chipping and delamination has made monolithic zirconia restorations a common alternative [5].

Monolithic zirconia is a full-contour restoration milled without veneering ceramic, usually as the final step in the laboratory process [6]. Owing to its uniform structure, the fracture resistance of a monolithic zirconia crown is greater than that of bilayered PFZ [4]. The elimination of veneering porcelain reduces the risk of chipping, allows for more conservative tooth preparation, and decreases both laboratory steps and costs, contributing to the growing popularity of monolithic zirconia restorations [7].

Both zirconia applications, whether as a coping material for FPDs or as full-contour monolithic restorations, often require chairside adjustments to achieve better fit, proper proximal contact, an ideal emergence profile, and optimal occlusal relationships [7,8]. However, there is no standardized protocol in the literature regarding the clinical adjustment of monolithic zirconia [2]. Various clinical methods for finishing and polishing, such as grinding with diamond burs, stone burs, or diamond-impregnated rubber tips, can be employed, often followed by the application of a glaze layer in the laboratory [5].

In PFZ restorations, veneering ceramics and luting cements protect the zirconia framework from direct exposure to the oral environment. In contrast, monolithic zirconia restorations are fully exposed to saliva, as well as to thermal and mechanical stresses. This condition facilitates the occurrence of low-temperature degradation (LTD) [1,6,9], a spontaneous and time-dependent transformation of the tetragonal phase into the monoclinic phase in zirconia when exposed to a humid environment at temperatures between 150 and 400 °C [10]. LTD is associated with grain pullout, microcracking, and strength reduction [8].

Clinical adjustment procedures typically use diamond burs due to zirconia’s high surface hardness. However, this can remove the glaze layer and significantly increase surface roughness, leading to wear of the opposing dentition, plaque accumulation, gingival inflammation, and changes in optical properties [2,5,8]. Surface alterations caused by adjustment may influence the mechanical properties and long-term performance of zirconia restorations in clinical use, including flexural strength, hardness, and elastic modulus.

Microcracks formed during grinding can serve as pathways for water penetration into the material, promoting LTD and mechanical degradation [10]. The literature presents conflicting results regarding the effect of adjustment on the strength of Y-TZP [10,11].

Flexural strength is a critical property of brittle materials and is commonly used as an indicator of strength [7,11]. Although many studies have examined the effects of various surface treatments on zirconia during chairside adjustments, none have specifically evaluated how the extent of adjustment affects zirconia strength after aging. The first null hypothesis was that the biaxial flexural strength (BFS) of monolithic zirconia would be significantly affected by the chairside adjustment procedure (grinding, polishing, or reglazing). The second null hypothesis was that the extent of adjustment (one vs. two areas) would not affect the BFS of monolithic zirconia after aging.

## Materials and methods

Materials

The material compositions used in this study are listed in Table 1.

**Table 1:** Materials, their main compositions, and manufacturers

Material	Composition (W%)	Trademark and manufacturer	Batch number
Pre-sintered zirconia blanks (yttrium partially stabilized zirconia)	ZrO₂: main component; Y₂O₃: 4.5-5.6; HfO₂ ≤ 5; Al₂O₃ ≤ 0.5; other oxides ≤ 1; flexural strength ≥ 800 MPa	Ceramill® ZI, White, Koblach, Austria: Amann Girrbach GmbH	2404001
Clear glaze	Glass of aluminum silicate with sodium solvents	Cerabien™ ZR, Tokyo, Japan: Kuraray Noritake Dental Inc.	EJQPQ
Zirconia diamond bur #ZF801L	106 grit; diamond fine; grit size 018; stainless steel	DiaTessin®, Switzerland: DiaTessin AG	10081
Polishing system (2-step)	Silicone elastomer matrix with diamond particles; green Sunburst: medium grit size; orange Sunburst: fine grit size	Diacera® RA-322, Germany: Eve Ernst Vetter GmbH	496807

Ethical approval and sample size calculation

The study protocol was approved by the Ethics Committee of Kerman University of Medical Sciences (IR.KMU.REC.1404.061). Based on ANOVA testing using G*Power software, with α = 0.05, power = 0.80, and an effect size of 0.55, the total sample size was determined to be 56 specimens (n = 8 per group) [6,12].

Preparation of specimen

In this experimental study, 56 standardized disk-shaped monolithic zirconia specimens (Ø15 × 1.2 ± 0.2 mm) were fabricated from pre-sintered zirconia blanks (Ceramill, Amann Girrbach, Koblach, Austria). The specimens were designed using computer-aided design software (Autodesk Inc., San Francisco, CA, USA). The design was transferred to computer-aided manufacturing software, and the position of the samples within the zirconia blocks was arranged accordingly. The pre-sintered disks were then milled using a 5-axis milling machine (Ceramill Motion 2, Amann Girrbach).

Considering approximately 20% shrinkage during sintering, the final dimensions of the specimens after sintering and polishing were about 15 mm in diameter and 1.2 ± 0.2 mm in thickness, according to ISO 6872:2024 [13]. The specimens were sintered for two hours at 1450 °C in a sintering furnace (Ceramill Therm 3, Amann Girrbach), following the manufacturer’s instructions. The temperature was increased at a rate of 3-10 °C/min to 1450 °C, then slowly cooled to room temperature at approximately 5 °C/min.

Next, the zirconia specimens were wet-finished with 600-grit and then sequentially polished with 800- and 1200-grit silicon carbide papers (Struers GmbH, Willich, Germany) using a polishing machine (Phoenix Beta Grinder/Polisher, Buehler, Lake Bluff, IL, USA). Each polishing step was performed for 15 seconds at 300 rpm under a 10 N load.

Since monolithic crowns are typically supplied in glazed form, all specimens were glazed. First, specimens were ultrasonically cleaned in 9% isopropyl alcohol for 10 minutes and air-dried. A glaze powder (Clear Glaze, Kuraray, Chiyoda, Japan) was mixed with its corresponding liquid and applied in a thin coat (approximately 0.05 mm) over the entire surface using a ceramic brush by an experienced dental technician [14]. Glaze firing was performed in a firing oven (Programat EP 3000, Ivoclar Vivadent, Schaan, Liechtenstein) according to the manufacturer’s instructions. The dimensions of each specimen were verified at three points using a digital caliper (Mitutoyo Corporation, Kawasaki, Japan).

Surface treatment

Each specimen was placed within a custom-made silicone matrix mold (Speedex, Coltene, Altstätten, Switzerland) to prevent movement during preparation and to ensure proper alignment between the specimen and the tool tip [7]. The specimens were randomly assigned to seven groups according to surface treatment type and extent of adjustment. The ground areas were defined in two sizes: “one area” (semicircle) and “two areas” (circle), corresponding to 21.5% and 43% of the total specimen surface, respectively.

The control group (G1) remained untouched after the sintering process. Finally, all groups were subjected to aging as follows: Group 1 (G1): control group (no treatment); Group 2 (G2): grinding one area; Group 3 (G3): grinding and polishing one area; Group 4 (G4): grinding and reglazing one area; Group 5 (G5): grinding two areas; Group 6 (G6): grinding and polishing two areas; and Group 7 (G7): grinding and reglazing two areas.

Grinding Procedure

To standardize the wear thickness and ensure that the entire surface was subjected to grinding, the specimens were marked with a permanent marking pen (Tombow ABT PRO, Tombow Pencil Co., Tokyo, Japan) [15]. Grinding was performed using a fine-grit (106 µm) blue-yellow band diamond bur (ZF801L, Dia-Tessin, Vanetti SA, Avegno Gordevio, Switzerland) attached to a high-speed contra-angle handpiece (Ti-Max X700, NSK, Shinagawa, Japan) with water coolant. The procedure was carried out in a forward-backward sweeping motion for one minute to simulate clinical conditions, using an equal number of strokes by a single trained operator (ZD). Grinding continued until all surface markings were completely removed (a level of movement control not typically achievable in a clinical setting). The diamond bur was replaced after every five specimens. After grinding, the specimens were inspected under direct illumination to confirm complete removal of the glaze layer, then ultrasonically cleaned in distilled water for 15 minutes and air-dried at room temperature.

Polishing Procedure

The ground surfaces of specimens in groups G3 and G6 were polished using an intraoral zirconia polishing kit (Diacera, EVE Ernst Vetter GmbH, Birkenfeld, Germany) in a two-step process with green (medium) and orange (fine) rubber polishers. Polishing was performed in the same sweeping direction as the grinding procedure for a total of two minutes (one minute per polisher and ground area) using a low-speed handpiece (NSK) [16]. The process was conducted with an equal number of strokes and consistent hand pressure by the same operator. Between the two polishing stages, the specimens were rinsed for 15 seconds with a water-air spray. The polishing instruments were replaced after every five specimens. After polishing, all specimens were ultrasonically cleaned in distilled water for 15 minutes and air-dried at room temperature.

Reglazing Procedure

Before reglazing, the ground areas were finished with a stone bur (SD716F.HP.150, Jota AG, Rüthi, Switzerland) by an experienced dental technician. The glazing material was then evenly applied to specimens in groups G4 and G7 and fired in a porcelain furnace according to the manufacturer’s instructions.

After each treatment, the dimensions of the specimens were rechecked at three points using a digital caliper to confirm compliance with the ISO range for the BFS test (Ø15 × 1.2 ± 0.2 mm).

Aging Procedure

To simulate the effects of LTD, the specimens were subjected to autoclave hydrothermal aging (E9 Med Class B, Euronda s.p.a., Sandrigo, Italy). They were placed on an autoclave tray in metal mesh baskets assigned to each group and exposed for five hours at 134 °C and 2 bar pressure (five cycles), following the ISO 13356:2015 standard [17]. One hour of this aging process is theoretically equivalent to three to four years of in vivo aging [18].

BFS

All specimens were stored in deionized water at 37 °C for 24 hours before testing. The BFS test was performed using a universal testing machine (Z050, ZwickRoell, Ulm, Germany) and the piston-on-three-balls method in accordance with ISO 6872 [13]. The disk specimens were centrally positioned on three steel balls (Ø = 3.2 mm) arranged in a triangular configuration 10 mm apart. A metallic piston (Ø = 1.4 mm) applied a load at the center of each specimen at a crosshead speed of 1 mm/min until fracture occurred. The treated surface of each specimen was placed in contact with the balls, thus experiencing tensile stress during testing. The maximum load at fracture was recorded.

The BFS (σ) was calculated according to ISO 6872 using the following equation:

Equation 1:

$\sigma = -0.2387 \cdot \frac{P}{d^2} \cdot (X - Y),$

where σ is the BFS in MPa, P is the maximum load at fracture (N), and d is the specimen thickness (=1.2 mm). X and Y were calculated using the following equations:

Equation 2:

\(X = (1 + \nu)\cdot \left[\ln{\left(\frac{r_2}{r_3}\right)}\right]^2 + \frac{1 - \nu}{2} \cdot \left(\frac{r_2}{r_3}\right)^2\)

Equation 3:

\(Y = (1 + \nu)\cdot \left[1 + \left(\ln{\left(\frac{r_1}{r_3}\right)}\right)^2\right] + (1 - \nu) \cdot \left(\frac{r_1}{r_3}\right)^2,\)

where ν is the poisson’s ratio of the ceramic (=0.25), r1 is the radius of the support ring (=5 mm), r2 is the radius of the loaded area (=0.7 mm), and r3 is the specimen radius (=7.5 mm).

Data analysis

Statistical analysis was conducted using IBM SPSS Statistics for Windows, Version 26.0 (Released 2018; IBM Corp., Armonk, NY, USA). The mean and SD of BFS values were calculated. Data normality was verified using the Kolmogorov-Smirnov test, confirming a normal distribution. A two-way ANOVA was performed to evaluate the effects and interaction of two factors, such as surface treatment and extent of adjustment, on BFS mean values. When statistically significant differences were detected, Tukey’s HSD post hoc test was applied for pairwise comparisons between the levels of each variable and among all experimental groups. The significance level was set at 0.05.

## Results

The mean and SD of BFS values for each group are shown in Table 2. Among all groups, G6 (grinding + polishing two areas) exhibited the highest mean BFS value (999.2 ± 81.13 MPa), whereas G4 (grinding + reglazing one area) showed the lowest (528.88 ± 17.54 MPa).

**Table 2:** Mean (SD) of BFS (MPa) in all experimental groups BFS, biaxial flexural strength

Group	Mean (SD)	Min	Max
G1 (control)	688.79 (25.22)	631.5	712.6
G2 (grinding one area)	781.83 (35.47)	738.6	839.6
G3 (grinding + polishing one area)	898.15 (76.93)	806.5	1034.6
G4 (grinding + reglazing one area)	528.88 (17.54)	495.8	551.7
G5 (grinding two areas)	829.50 (76.29)	710	943.6
G6 (grinding + polishing two areas)	999.20 (81.13)	849.8	1115.4
G7 (grinding + reglazing two areas)	657.36 (18.45)	631.3	678.5

The two-way ANOVA results indicated that both surface treatment and extent of adjustment had significant effects on BFS (p = 0.0001). However, the interaction between these two factors was not statistically significant (p = 0.114) (Table 3).

**Table 3:** Effects of surface treatment and extent of adjustment on BFS BFS, biaxial flexural strength

Source	Type III sum of squares	Mean square	F	p-Value
Surface treatment	1,024,453.670	512,226.835	171.864	0.0001
Extent of adjustment	102,517.810	102,517.810	34.397	0.0001
Surface treatment × extent of adjustment	13,515.025	6,757.513	2.267	0.114

**Figure 1:**
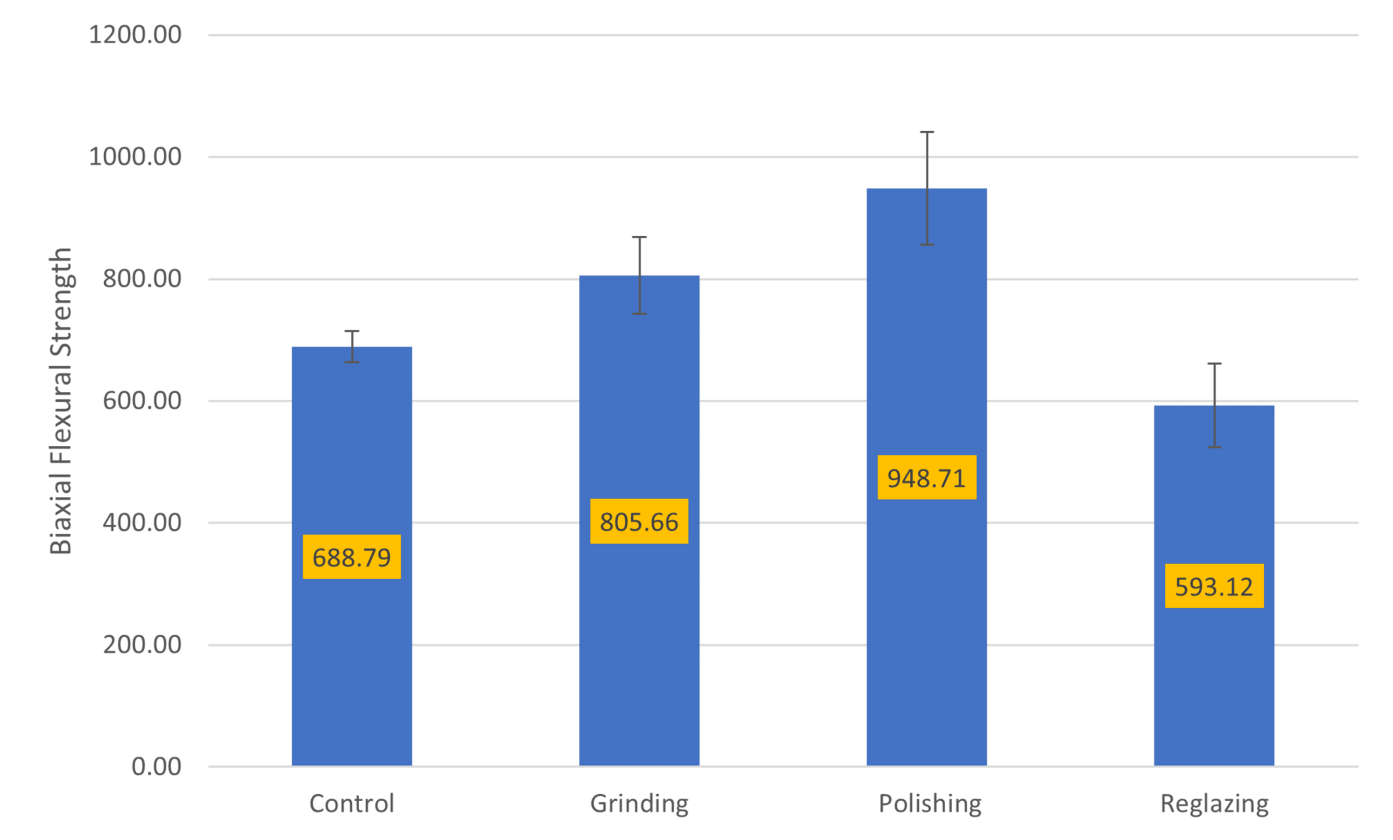
Pairwise comparison of BFS based on surface treatment BFS, biaxial flexural strength

As shown in Figure 1, specimens subjected to polishing demonstrated the highest mean BFS values, while reglazed specimens exhibited significantly lower values, regardless of the extent of adjustment.

Pairwise comparisons among surface treatment groups revealed significant differences in BFS values for group (Table 4).

**Table 4:** Pairwise comparisons of BFS based on surface treatment BFS, biaxial flexural strength

Group	n	Mean (SD)	p-Value
Control (C)	8	688.78 (25.22)	C and G = 0.0001, C and P = 0.0001, C and R = 0.0001
Grinding (G)	16	805.66 (62.52)	G and P = 0.0001, G and R = 0.0001
Polishing (P)	16	948.71 (92.52)	P and R = 0.0001
Reglazing (R)	16	593.11 (68.59)	—

**Figure 2:**
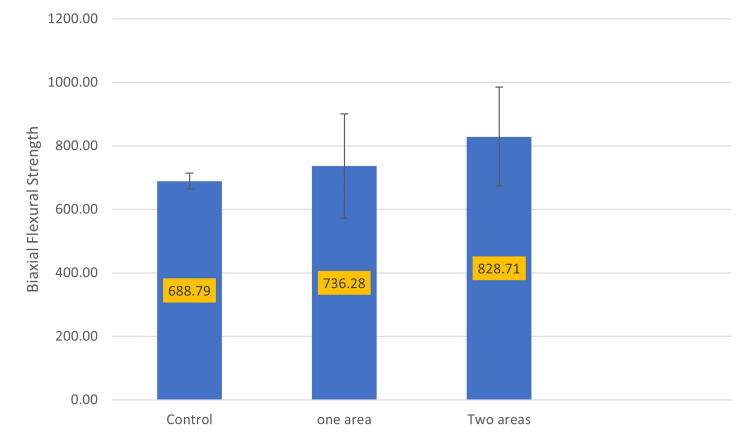
Pairwise comparison of BFS based on extent of adjustment BFS, biaxial flexural strength

Regardless of surface treatment, specimens with two adjusted areas exhibited significantly higher BFS values than those with one adjusted area or no adjustment, as shown in Figure 2.

Pairwise comparisons for the extent of adjustment (Table 5) demonstrated that groups with two adjusted areas (G5, G6, and G7) had significantly higher BFS values compared with both the control and one-area adjustment groups (G2, G3, and G4) (p = 0.0001). A marginally significant difference was observed between the control and one-area adjustment groups (p = 0.09).

**Table 5:** Pairwise comparisons of BFS based on extent of adjustment BFS, biaxial flexural strength

Group	n	Mean (SD)	p-Value
Control (C)	8	688.78 (25.22)	C and O = 0.09, C and T = 0.0001
One area (O)	24	736.28 (164.54)	O and T = 0.0001
Two areas (T)	24	828.71 (155.59)	—

Multiple pairwise comparisons among all experimental groups are summarized in Table 6. Significant differences (p < 0.05) were observed across most groups, except between G5 (grinding two areas) and G2 (grinding one area) (p = 0.58), between G5 (grinding two areas) and G3 (grinding + polishing one area) (p = 0.17), and between G7 (grinding + reglazing two areas) and the control group (p = 0.90).

**Table 6:** Pairwise comparisons between all experimental groups for the combined effects of surface treatment and extent of adjustment ^*^ The mean difference is significant at the 0.05 level.

(I) group	(J) group	Mean Difference (I-J)	p-Value	95% CI	
				Lower bound	Upper bound
G1	G2	-93.03750^*^	0.02	-176.948	-9.1267
	G3	-209.36250^*^	0.0001	-293.273	-125.452
	G4	159.91250^*^	0.0001	76.0017	243.8233
	G5	-140.71250^*^	0.0001	-224.623	-56.8017
	G6	-310.48750^*^	0.0001	-394.398	-226.577
	G7	31.425	0.9	-52.4858	115.3358
G2	G3	-116.32500^*^	0.002	-200.236	-32.4142
	G4	252.95000^*^	0.0001	169.0392	336.8608
	G5	-47.675	0.58	-131.586	36.2358
	G6	-217.45000^*^	0.0001	-301.361	-133.539
	G7	124.46250^*^	0.001	40.5517	208.3733
G3	G4	369.27500^*^	0.0001	285.3642	453.1858
	G5	68.65	0.17	-15.2608	152.5608
	G6	-101.12500^*^	0.009	-185.036	-17.2142
	G7	240.78750^*^	0.0001	156.8767	324.6983
G4	G5	-300.62500^*^	0.0001	-384.536	-216.714
	G6	-470.40000^*^	0.0001	-554.311	-386.489
	G7	-128.48750^*^	0.0001	-212.398	-44.5767
G5	G6	-169.77500^*^	0.0001	-253.686	-85.8642
	G7	172.13750^*^	0.0001	88.2267	256.0483
G6	G7	341.91250^*^	0.0001	258.0017	425.8233

## Discussion

This in vitro study aimed to evaluate the effect of the extent of chairside adjustment on the BFS of monolithic zirconia. Although the use of mechanical grinding and polishing devices allows for standardization and reproducibility, the surface defects they produce differ from those created by diamond burs in clinical practice. To better replicate clinical conditions, a chairside grinding and polishing protocol was applied in this study [15,19].

Despite some inconsistencies in the literature regarding the impact of LTD on the mechanical properties of zirconia, many studies have reported that LTD reduces the strength of zirconia and increases its surface roughness [20]. LTD simulation is commonly performed using autoclave treatment [18]. In this study, all groups were subjected to LTD to closely mimic oral conditions. As supported by previous reports, autoclaving zirconia at 120-140 °C effectively induces hydrothermally activated phase transformation, serving as a reliable method for accelerated LTD simulation [21].

The toughness of zirconia primarily depends on its tetragonal-to-monoclinic (t→m) phase transformation. This transformation can either enhance or weaken its mechanical properties, depending on the extent to which it occurs. The approximately 4% volumetric expansion associated with this transformation leads to compressive stresses that hinder crack propagation, a mechanism known as transformation toughening. In addition, other mechanisms such as crack deflection, crack bridging, and crack tip shielding collectively contribute to zirconia’s resistance to fracture by reducing the driving force for crack propagation. However, excessive transformation to the monoclinic phase can be detrimental, as this phase possesses inferior mechanical properties compared to the tetragonal phase [8,22].

In the present study, even the lowest mean BFS value (observed in group G4) exceeded 500 MPa, which is higher than the average occlusal load typically experienced during mastication [23]. This indicates that, although surface treatments influenced strength, all zirconia specimens maintained sufficient mechanical integrity for clinical use.

Overall, the results of this study demonstrate that the adjustment protocol, including grinding, polishing, and reglazing, significantly affected the BFS of monolithic zirconia. Therefore, the first null hypothesis was rejected.

Effect of grinding

In this study, BFS values increased after grinding, which aligns with the findings of previous studies [24,25]. However, other reports have shown either decreases [26-28] or no significant changes in strength following grinding [21,29]. The observed increase in flexural strength can be attributed to the tetragonal-to-monoclinic (t→m) phase transformation that occurs during grinding. This transformation results in a volumetric expansion and shear strain near the crack tip, producing a thin compressive stress layer (approximately 15-20 µm) that counteracts crack propagation and mitigates the negative effects of LTD [19,22,30].

During the chairside adjustment of zirconia restorations, the glazed surface is often subjected to grinding either before or after cementation [6]. Grinding can introduce surface flaws and cracks, increasing surface roughness and allowing greater water penetration into the bulk material, which in turn makes zirconia more susceptible to LTD [19]. Increased surface roughness can also negatively influence flexural strength, optical properties, wear of opposing dentition, and biofilm accumulation [8]. Although surface roughness is generally inversely correlated with flexural strength, this relationship depends on the relative depth of grinding-induced defects compared with preexisting surface flaws [31]. A stronger correlation is observed when grinding-induced cracks extend beyond the depth of existing flaws and the compressive stress layer [15]. When the crack depth exceeds this compressive layer, the flexural strength of zirconia may decrease [24,29]. Therefore, the overall mechanical performance of ground zirconia depends on the balance between the beneficial effects of transformation toughening and the detrimental effects of critical surface defects [6,19,30].

The inconsistent findings in the literature regarding the influence of grinding on zirconia strength may be attributed to differences in study methodologies, as several variables can affect outcomes [32]. Grinding parameters, such as grit size, type of grinding tool, presence or absence of coolant, applied pressure, and duration, can all influence the extent of phase transformation and the mechanical behavior of zirconia [19]. For example, Ho et al. reported that using a diamond bur increased flexural strength, while Lee et al. found that coarse diamond burs reduced strength due to the formation of surface flaws and microcracks [9,33]. Grinding with fine grit size (≤50 µm), low-speed handpieces, contra-angle attachments, and consistent coolant use tends to minimize defect formation and promote transformation toughening [19]. In contrast, grinding without coolant can raise surface temperatures above the critical threshold, potentially causing undesirable t→m transformation or even reverse transformation, as reported by Kosmac et al., who observed a reduction in monoclinic phase content and overall strength [34].

In the present study, a fine diamond bur and a two-step polishing system were used under continuous air and water coolant. These conditions likely prevented excessive temperature rise, thus avoiding any reverse phase transformation or strength reduction [6]. Furthermore, material-specific characteristics such as grain size and zirconia composition also play crucial roles in determining the final mechanical properties [19,35].

Effect of polishing

The surface roughness produced by chairside grinding can be removed either through glazing or polishing [36]. Bur abrasion can cause grain pull-out and microcrack propagation, which in turn leads to mechanical deterioration and hydrothermal degradation of zirconia [3,37]. Chairside polishing eliminates the defects generated during surface grinding, reduces surface roughness, and improves resistance to aging [35,38,39]. It also provides a practical advantage by eliminating the need to return the restoration to the laboratory, thereby saving time and avoiding additional patient appointments [38]. Intraoral polishing helps counteract the negative effects of grinding and can partially restore the smoothness of zirconia surfaces [2,5,35]. A poorly polished zirconia surface may compromise the durability and longevity of the restoration, whereas a well-polished surface enhances surface integrity and causes less wear on opposing dentition compared with glazed surfaces [3,40].

The results of this study showed an increase in the BFS of ground zirconia after polishing, which agrees with previous findings [7,11,12,16]. Polishing generally does not induce significant phase transformation in zirconia [16,37]. Fine polishing can reduce the monoclinic phase content of ground zirconia, thereby removing compressive stresses and minimizing surface shear strain [11,16,22]. A surface stress layer forms after grinding and polishing. If the cracks induced by grinding are confined to this superficial compressive layer, no significant difference in strength after polishing may be observed [37]. However, in most cases, optimal polishing removes material beyond the depth of the transformed layer, which can influence strength outcomes [25,26].

Contrary to the findings of this study, Guazzato et al. and Pittayachawan et al. reported that polishing reduced the flexural strength of zirconia; however, their polishing protocols were not representative of common clinical practice [25,41].

The homogeneous crystalline structure of zirconia contributes to its superior polishability compared to glass ceramics and feldspathic porcelain. In contrast, multiphasic ceramics, where crystalline fillers are exposed after removing the weaker glassy phase, can be more difficult to polish effectively. Given zirconia’s high hardness (exceeding 1200 HVN), conventional porcelain finishing and polishing systems are often inadequate, necessitating advanced polishing systems designed specifically for zirconia [5]. Effective zirconia polishing protocols typically involve two or three sequential polishing steps, starting with a coarse tool and progressing to a fine one to gradually reduce surface roughness [35]. The performance of a polishing system depends on factors such as abrasive particle type (natural or synthetic diamond), shape, grain size, density, and binder material. Diamond-based systems have been shown to outperform silicon carbide systems in reducing zirconia surface roughness, owing to the higher hardness of diamonds [40].

In this study, we used the EVE Diacera intraoral zirconia polishing kit, which consists of synthetic rubber burs embedded with diamond particles. Previous reports indicate that this system produces smoother and more uniform zirconia surfaces. Polishing with finer diamond particles has been associated with reduced residual stress and higher flexural strength, supporting the superiority of zirconia-specific polishing kits [2,36].

According to the literature, rubber-based polishing systems appear to be the preferred method for zirconia in terms of achieving both surface smoothness and fracture resistance [2,7]. de Carvalho et al. found that rubber polishing promotes compression of zirconia grains at the surface, thereby reducing crack propagation and increasing material strength [3]. Similarly, Mohammadi-Bassir et al. reported enhanced flexural strength when using rubber burs [11].

Effect of reglazing

Glazing is a common method used to smooth the surface of zirconia and enhance its optical properties [42]. It provides a lustrous finish and natural tooth-like appearance [14]. However, Lai et al. [43] reported that although the glaze layer may initially protect zirconia from LTD, disintegration of the glaze layer can occur within the first six months of clinical service [44]. Moreover, reglazing can obscure surface details and texture, cause noticeable color changes in restorations [45], and contribute to significant wear of opposing teeth. This wear is attributed to the low fracture toughness of the glaze layer, which fractures into small particles and induces three-body abrasion [46].

In this study, reglazing after grinding led to a decrease in BFS, consistent with other reports [7,11,14,25,32,42,47]. This reduction in strength may result from crack formation associated with repeated firings [8]. Cracks tend to propagate from the glaze layer toward the zirconia, suggesting that fracture of glazed monolithic zirconia typically initiates in the glaze [42]. Furthermore, high-temperature exposure during glazing may promote reverse phase transformation from monoclinic to tetragonal (m→t), leading to loss of the protective compressive stress layer and weakening of the material [5,11,47].

Because Y-TZP is a polycrystalline material with minimal glass content, bonding to the glaze layer can be challenging. In this bilayered system, the tensile material, the glaze layer, largely determines the mechanical behavior, especially when adhesion between layers is poor. Fracture frequently occurs at the zirconia-glaze interface, and factors such as glaze layer thickness, bubbles, cracks, and incomplete coverage can further reduce strength. In the present study, these issues were minimized by adhering to a standardized glazing protocol [39,48].

Contrary to our findings, some studies have reported increased BFS after reglazing [12,49,50]. Chougule and Wadkar suggested that reglazing may improve flexural strength by filling surface flaws and blunting flaw tips, following Anusavice’s explanation of the crack inhibition phenomenon, thus increasing overall strength [49,51].

Effect of extent of adjustment

The results of this study demonstrated that the extent of adjustment significantly influenced the BFS in the grinding, polishing, and reglazing groups, leading to rejection of the second null hypothesis. Higher BFS values observed in the two-area grinding and polishing groups compared to their one-area counterparts (though not statistically significant for grinding) may be attributed to differences in the extent of phase transformation and monoclinic phase content. These factors influence zirconia’s ability to counteract crack propagation through the transformation toughening mechanism and affect the fatigue behavior of restorations during service.

During chairside adjustment, it is important to consider that zirconia restorations have already undergone multiple mechanical and thermal stresses during industrial processing and laboratory fabrication. Excessive or repeated phase transformations may lead to the degradation of physical properties through the formation of cracks and internal stresses [52]. While the volume expansion and compressive stresses associated with the tetragonal-to-monoclinic transformation can enhance the material’s durability, uncontrolled transformation can cause cracking and eventual failure [53]. The extent of this transformation is influenced by both material characteristics and surface treatment procedures, including the instruments and methods used [22,52].

Carefully controlled grinding conditions for partially stabilized zirconia can improve surface integrity and flexural strength [54]. A gentle grinding and polishing protocol, characterized by minimal temperature rise and the use of fine and superfine rubber polishers with controlled strokes under water coolant, may explain the higher BFS values observed with increased adjustment extent [55]. Proper fine finishing and polishing procedures can substantially mitigate the negative effects of phase transformation and significantly enhance fracture resistance [37].

In this study, reglazing one area resulted in significantly lower flexural strength compared to reglazing two areas. This finding may be due to the fact that polishing is typically limited to chairside ground areas, whereas reglazing often covers all external restoration surfaces, leading to an increased glaze layer thickness. Nonaka et al. reported that flexural strength decreases with increasing glaze thickness, which is operator dependent but typically around 50 µm [42]. During flexural testing, the brittle glaze layer may fracture before the underlying zirconia, and detached glaze chips may accelerate failure. Moreover, crevices or flaking in the glaze layer can expose zirconia to LTD [42,43]. Reduction in BFS is also associated with fracture initiation at voids within the glaze layer, caused by mismatched coefficients of thermal expansion between the glaze and zirconia at the interface [14,47].

It is important to note that even minor differences in composition, manufacturing, or sintering among Y-TZP ceramics can alter their susceptibility to transformation and LTD [1]. Therefore, one limitation of this study is the use of only one brand of zirconia, which may limit the generalizability of the findings to other formulations. Another limitation is the inability to standardize the pressure and force applied during grinding and polishing, which reflects the inherent variability of clinical procedures. Furthermore, since the fatigue behavior of zirconia may differ from the outcomes of monotonic strength tests [30], this study did not assess long-term stability under cyclic loading conditions. Future investigations integrating advanced characterization techniques (X-ray diffraction, scanning electron microscopy, and Raman) and computational modeling (finite element analysis) could provide a more detailed understanding of t→m transformations, subsurface damage, residual stresses, and their evolution under functional loading. Clinically, optimizing laboratory processes, such as design, milling, and polishing, remains essential to minimize or eliminate the need for extensive chairside adjustments during the delivery of zirconia restorations.

## Conclusions

Within the limitations of this in vitro study, it can be concluded that both the surface treatment and the extent of adjustment influenced the BFS of monolithic zirconia. Rubber polishing significantly enhanced the BFS of ground zirconia, whereas reglazing markedly reduced it. Increasing the extent of grinding and polishing further improved BFS, while extending the area of reglazing amplified its adverse effect on strength.
